# A Non-traumatic Non-aneurysmal Subarachnoid Hemorrhage in a Mild COVID-19 Infection: A Case Report

**DOI:** 10.7759/cureus.34103

**Published:** 2023-01-23

**Authors:** Mohammad Abu-Abaa, Ali Abdulsahib, Ghassan Al-Qaysi, Hassaan Arshad

**Affiliations:** 1 Internal Medicine, Internal Medicine Residency Program, Capital Health Regional Medical Center, Trenton, USA; 2 Internal Medicine, Capital Health Regional Medical Center, Trenton, USA

**Keywords:** bleeding, intracranial hemorrhage (ich), headache, spontaneous subarachnoid hemorrhage, covid 19

## Abstract

Both subarachnoid hemorrhage and intraparenchymal hemorrhage have been reported in patients with coronavirus disease 2019 (COVID-19) infection. We report a 38-year-old male patient who was initially admitted for alcoholic hepatitis and had a mild COVID-19 infection that was confirmed 10 days prior to presentation. During his hospitalization, he reported worsening of his occipital headache that started when he tested positive for COVID-19. Neurological examination was intact and no history of trauma, hypertension, illicit drug use, or family history of brain aneurysm was reported. Investigating his worsening headache revealed a tiny, right-sided, posterior subarachnoid hemorrhage. No coagulopathy was evident. No aneurysm was seen on the cerebral angiogram. The patient was managed conservatively. This case raises the point of the importance of investigating headaches even in mild COVID-19 infection, as it may herald intracranial bleeding.

## Introduction

Many cases of subarachnoid hemorrhage (SAH) in those with coronavirus disease 2019 (COVID-19) infection were reported in the English literature [1-4]. SAH accounts for 1-6% of all stroke cases and around 10-15% of all patients die before presentation to a hospital [5]. A common finding in the vast majority of COVID-19-related cases of SAH is severe COVID-19 infection and mortality [6-8]. In our patient, the common etiologies of SAH, including trauma, hypertension, illicit drug use, and coagulopathies, were ruled out. Our case differs, as COVID-19 was mild and the patient was being treated for another issue but persistent headache prompted further investigation. This raises the clinical pearl of having a low threshold to investigate headaches in those with COVID-19 infection regardless of the severity.

## Case presentation

A 38-year-old male patient presented with worsening right upper abdominal quadrant (RUQ) pain. Past medical history is remarkable for alcohol use disorder, alcoholic pancreatitis, and hepatitis. He was not taking any medication at home. He denied a history of hypertension, smoking, illicit drug use, excessive alcohol use, and a family history of subarachnoid hemorrhage. He did not receive a COVID-19 vaccine. He tested positive for COVID-19 infection 10 days prior to this presentation. In the emergency department (ED), vital signs included a temperature of 37.3 degrees Celsius, blood pressure of 125/75 mmHg, respiratory rate of 22 cycles per minute, heart rate of 78 beats per minute, and oxygen saturation (SpO2) of 96% on room air. A physical exam showed a distended abdomen with RUQ tenderness and was otherwise unremarkable. The neurological examination was unremarkable. The urine drug screen was negative. Basic lab work was significant for direct hyperbilirubinemia, and the patient was admitted for alcoholic hepatitis.

**Figure 1:**
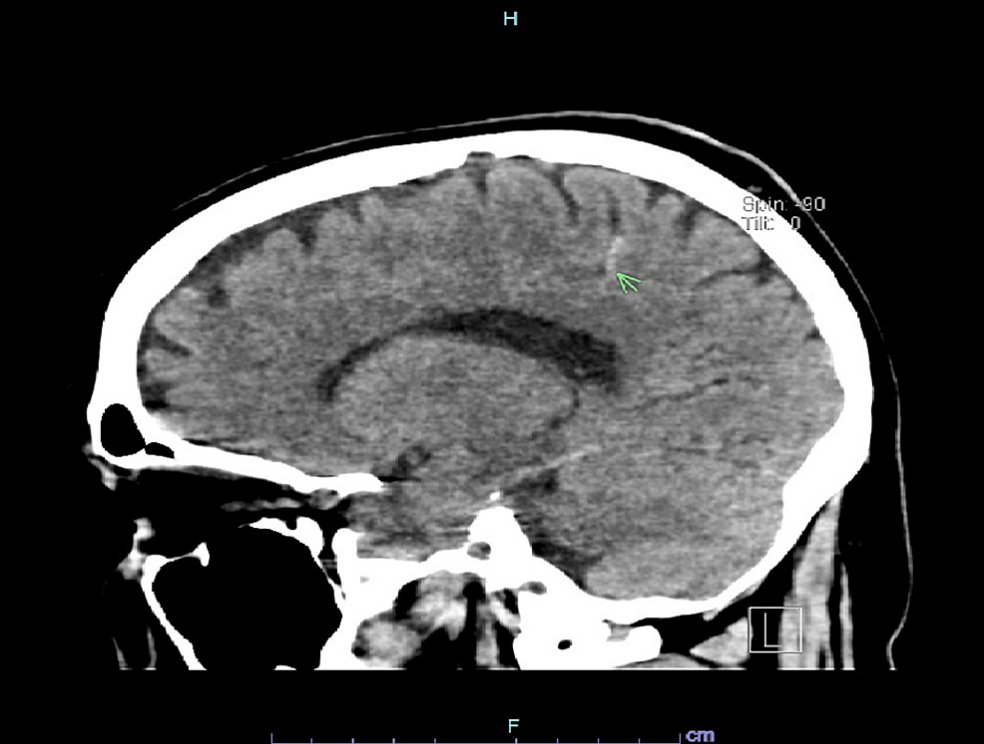
CT Head CT head showing evidence of subarachnoid hemorrhage (arrow)

He tested positive on COVID-19 polymerase chain reaction (PCR). C-reactive protein (CRP) was elevated at 3.5 mg/dl (reference less than 1 mg/dl) with elevated ferritin level at 754 ng/ml (reference 17-464 ng/ml), elevated LDH at 579 U/L (reference 120-246 U/L), and elevated D-dimer at 1.27 mcg/ml (reference 0-0.45 mcg/ml). The patient did not require an oxygen supply y throughout his hospitalization. The coagulation profile, including prothrombin time (PT), partial thromboplastin time (PTT), and international normalized ratio (INR) were within normal limits. On the second day of his hospitalization, he started to complain of a worsening episodic headache on standing. He admitted that the headache started around the same time he tested positive for COVID-19 10 days prior to admission. The headache was mainly occipital and associated with light sensitivity. He denied a history of trauma from a fall. He denied a history of illicit drug use. The neurological examination remained intact. Computed tomography (CT) scan of the head showed a tiny linear hyperdensity in the subcortical white matter of the right posterior parieto-occipital lobe (Figure 1).

**Figure 2:**
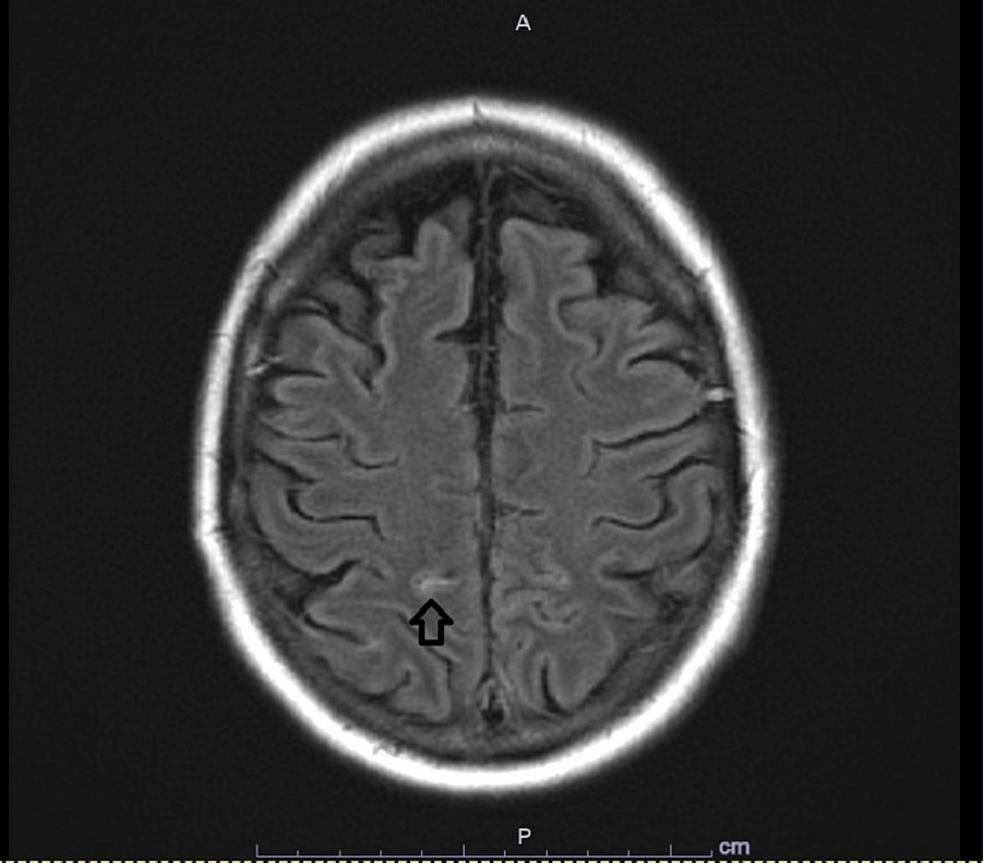
MRI Brain MRI fluid attenuation inversion recovery (FLAIR) brain showing evidence of subarachnoid hemorrhage (arrow)

**Figure 3:**
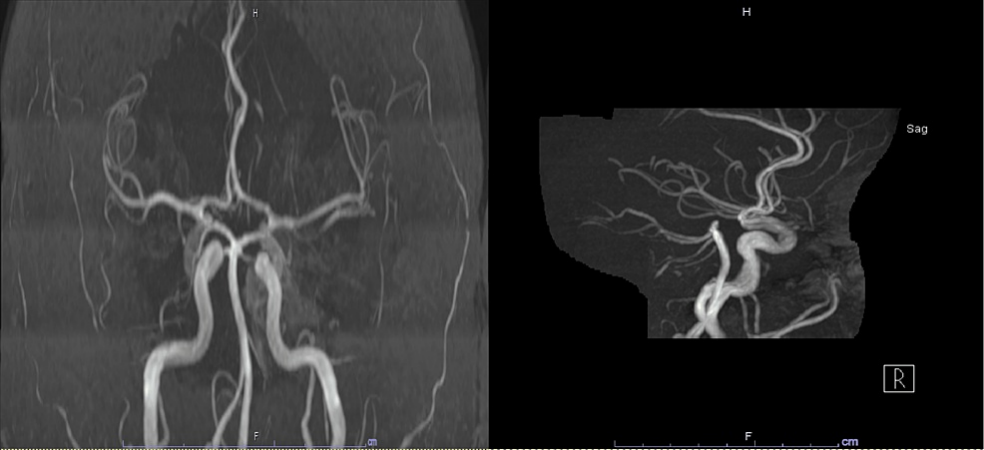
MRA Brain and Neck MRA brain and neck showing no evidence of aneurysm of arterio-venous malformation

Magnetic resonance imaging (MRI) brain showed focal fluid attenuation inversion recovery (FLAIR) hyperintensity in the right anteromedial parietal lobe (Figure 2). Magnetic resonance angiography (MRA) brain and neck was unremarkable for any aneurysm (Figure 3). The cerebral angiogram was also negative for any vascular abnormality.

The patient was managed symptomatically and symptoms resolved within a few days, allowing for discharge.

## Discussion

The association between COVID-19 infection and both aneurysmal and non-aneurysmal subarachnoid hemorrhage (SAH) has been suggested [1]. Around 85% of cases of SAH are traumatic and around 10% of cases have no identifiable source of bleeding [9]. The incidence of SAH among COVID-19 patients is estimated at 0.3% to 1.2% [3]. A case-control study of 247 patients with both COVID-19 and SAH in Japan concluded that SAH tends to be seen at a younger age in males compared to females [10].

Although the most accurate diagnostic test of aneurysmal SAH is a cerebral angiogram, it can be false-negative in 10-15% of cases, especially in perimesencephalic SAH [11]. Multiple reasons for false negative cerebral angiogram exist, including very small aneurysm, proximal and distal vasoconstriction impairing aneurysmal filling, intra-aneurysmal thrombus, cisternal blood hiding the aneurysm, and close proximity of bone impairing the image [11]. The fact that, in this case, no aneurysm was seen raises the question of the extent of the association between SAH and COVID-19.

COVID-19 infection is known to cause vascular endothelial dysfunction, which increases the risk of rupture, especially at aneurysmal sites [12]. This can be attributed to the excess local production of anti-proteases and oxygen free radicals by leukocytes and hypercoagulability by the release of tissue factor [13]. This direct endothelial toxicity leads ultimately to increased permeability, and disruption of cellular tight junctions and the blood brain barrier, which leads to an increased tendency for intracranial bleeding, including SAH. Also, as the virus itself suppresses cellular expression of angiotensin type-2 receptors, this leads to cellular accumulation of angiotensin type 2 and disruption of cerebral flow regulation, which also contributes to bleeding tendency [14].

Cezar-Junior et al. reported a case series of four cases of COVID-19 infection associated with SAH. Only two cases were non-aneurysmal. However, a significant elevation of inflammatory markers was seen in those cases, a median elevation of CRP of 3835 mg/dl, and a median elevation of D-dimer of 2336 [1]. Batcik et al. also reported four cases of SAH in association with COVID infection. Aneurysm was found in only one of them and all patients died. In these cases, SAH happened both early and late in the course of infection [2]. A large European multicenter study reported 18 cases of intracranial hemorrhage in association with COVID-19. Only 11 of them were SAH, and 10 of them were non-aneurysmal [15]. Melegari et al. reported two cases of SAH in patients who recovered after the development of severe COVID-19 infection and acute hypoxemic respiratory failure [16]. Other case series and reports of severe COVID-19 infection and SAH were also reported and mostly resulted in the patient's demise [7,10-12]. In contrast to all of these cases with severe COVID-19 infection, COVID-19 infection in the current patient was mild, given the lack of pulmonary manifestation as well as mildly elevated inflammatory markers. This serves the purpose of this case report, which is to remind clinicians of keeping a high level of awareness of the neurological complications of COVID-19 infection regardless of the severity

## Conclusions

Headache is a very non-specific symptom and is common among hospitalized patients. However, its occurrence in COVID-19 patients should always be investigated, even if the COVID-19 infection is not severe. Both intraparenchymal hemorrhage and SAH were reported in higher frequency among COVID-19 patients than in the general population.
